# DNA-PKcs participates in the repair of renal tubular epithelial cell injury

**DOI:** 10.1080/0886022X.2025.2537811

**Published:** 2025-07-29

**Authors:** Dan Feng, Yu-Jie Hu, Lei Sun, Jing Zhang, Xiao-Ling Niu, Wen-Yan Huang

**Affiliations:** Department of Nephrology and Rheumatology, Shanghai Children’s Hospital, School of medicine, Shanghai Jiao Tong University, Shanghai, China

**Keywords:** DNA-PKcs, DNA-PKcs inhibitor, renal tubular epithelial cell injury, repair

## Abstract

Ischemia/reperfusion (I/R) injury is a major cause of acute kidney injury (AKI) and plays a central role in mediating cell damage and ultimately acute tubular necrosis. Renal proximal tubular epithelial cells (PTECs) possess intrinsic repair mechanisms, yet the molecular pathways underpinning their ability to recover after I/R injury remain incompletely understood. DNA-dependent protein kinase catalytic subunit (DNA-PKcs), a serine/threonine kinase, is pivotal in DNA damage repair, genomic stability, cell cycle regulation, and mitochondrial dysfunction. Given its central roles in maintaining cellular homeostasis, we hypothesized that DNA-PKcs is critically involved in orchestrating the intrinsic repair mechanisms of renal tubular epithelial cells following I/R injury. In this study, we investigate the involvement of DNA-PKcs in the repair of tubular epithelial cell damage induced by renal I/R injury. Using both *in vitro* and *in vivo* models, we demonstrate that DNA-PKcs expression is significantly upregulated during the acute phase of kidney injury and returns to baseline levels upon resolution of the damage. In the hypoxia/reoxygenation (H/R) model using NRK-52E cells, treatment with the DNA-PKcs inhibitor NU7441 resulted in mitochondrial swelling. Additionally, the expression levels of DNA damage and epithelial-mesenchymal transition markers such as γ-H2AX, α-SMA, and vimentin were notably prolonged. Moreover, DNA-PKcs inhibition significantly impaired cell proliferation, induced a G1/S phase arrest under normoxic conditions, and resulted in G2/M phase arrest following H/R. Our study provides that DNA-PKcs acts as a promising therapeutic target for mitigating AKI and promoting renal regeneration.

## 1. Introduction

Acute kidney injury (AKI) is characterized by a rapid decline in renal function due to a variety of etiological factors. Ischemia-reperfusion (I/R) injury is one of the most common causes of AKI. Ischemia/reperfusion (I/R) injury refers to the damage caused during the period of blood supply deprivation (ischemia) and the subsequent recovery of blood supply (reperfusion) in an organ or tissue. During the reperfusion process, oxygen rapidly reenters the ischemic area, leading to the production of a large number of reactive oxygen species (ROS) and reactive nitrogen species (RNS). These free radicals can directly damage cell membranes, proteins, and DNA, thereby triggering cell apoptosis or necrosis [1–5]. Proximal tubular epithelial cells are the main targets of AKI [2,3]. Despite being highly susceptible to various forms of stress, these cells possess intrinsic repair mechanisms that enable them to minimize damage and prevent severe renal dysfunction [6]. Renal tubular repair can result in three distinct cellular outcomes [1]: (1) moderate repair, where both tubular structure and function are fully restored to normal; (2) excessive repair, where cell proliferation and excessive extracellular matrix synthesis contribute to renal fibrosis; and (3) delayed repair, where the damage persists. Understanding the mechanisms underlying tubular repair after injury is of great clinical significance, as it may help mitigate cellular damage, promote the timely entry of cells into the appropriate repair cycle, and restore their normal structure. Moreover, such research could identify potential therapeutic targets for AKI intervention, thereby improving prognosis and reducing mortality.

DNA-dependent protein kinase catalytic subunit (DNA-PKcs) is a serine/threonine kinase that contains a PI3K-binding domain at its C-terminal. It is a member of the PI3K family and shares structural similarities with ataxia-telangiectasia mutated (ATM) and ataxia-telangiectasia and Rad3-related (ATR) proteins. DNA-PKcs plays a pivotal role in DNA damage repair, maintaining genomic stability, regulating the cell cycle, and triggering mitochondrial dysfunction [7–12]. Most studies on DNA-PKcs have focused on cancer [13]; however, an increasing body of evidence suggests that DNA-PKcs also contributes significantly to the progression of various diseases, including aging, pulmonary fibrosis, and hypoxic pulmonary arterial hypertension [8,12,14,15]. Recent studies in the field of nephrology have examined the role of DNA-PKcs in both AKI and chronic kidney disease (CKD) [16,17]. Conditional knockout of DNA-PKcs in renal tubular epithelial cells has demonstrated that DNA-PKcs promotes the development of both AKI and CKD. However, research on the role of DNA-PKcs in renal tubular repair following AKI remains scarce. Given its central roles in maintaining cellular homeostasis, we hypothesized that DNA-PKcs is critically involved in orchestrating the intrinsic repair mechanisms of renal tubular epithelial cells following I/R injury.This research aims to explore how DNA-PKcs participates in the repair of renal tubular epithelial cell injury, with the hope of providing novel insights into potential strategies for enhancing the repair of AKI.

## 2. Materials and methods

### 2.1. Cell culture and H/R model

Rat proximal tubular cells (NRK-52E, CRL-1571) were purchased from the American Type Culture Collection. The cells were cultured in Dulbecco’s Modified Eagle Medium (DMEM, Gibco) containing 10% fetal bovine serum (FBS, Gibco) in an incubator with 5% CO_2_ at 37 °C.

The hypoxia/reoxygenation (H/R) model was established as follows: after the cells attached and reached 70%–80% confluence, the culture medium was replaced with a serum-free medium. At the same time, mineral oil (less dense than the culture medium and insoluble in water; Sigma) was added to isolate the cells from the air for 3 h[18]. After the hypoxia treatment, the mineral oil and serum-free-DMEM were removed, and the cells were flushed with phosphate-buffered saline (PBS). The medium was replaced with fresh DMEM containing 10% FBS. The cells were cultured in a regular incubator for reoxygenation for 0, 3, 6, 12 and 24 h. At the same time, a control group without any treatment was established.

### 2.2. Inhibition of DNA-PKcs function

NU7441 (8-dibenzothiophen-4-yl-2-morphin-4-yl-chromen-4-one, Tocris), a potent and selective DNA-PK inhibitor, was dissolved in DMSO and stored at −20 °C in a freezer. Cells were pretreated with 1 µM NU7441 2 h before H/R to inhibit the function of DNA-PKcs.

### 2.3. Western blot analysis

NRK-52E cells were washed twice with PBS and subjected to protein extraction by using radioimmunoprecipitation buffer (Beyotime) containing a phosphatase inhibitor (Roche) and protease inhibitor (Roche) for 30 min on ice. Cell lysates were centrifuged (12,000 rpm, 15 min) at 4 °C and supernatants were collected. A pair of sterile scissors was used to cut 100 mg of kidney tissue that had been stored at −80 °C. The tissue was placed in a sterile EP tube and added with the same lysate that was added to the extracted cell protein. An ultrasonic instrument was used to lyse tissue on ice to extract protein. Centrifugation (12,000 rpm, 15 min) at 4 °C and supernatant collection were then performed. Protein concentration was measured through the bicinchoninic acid method by using a protein assay reagent kit. Cell lysates (20 μg) were resolved *via* sodium dodecyl sulfate–polyacrylamide gel electrophoresis and transferred to PVDF membranes. The membranes were blocked with 5% skimmed milk (Bio-Rad) for 60 min and incubated with primary antibodies overnight at 4 °C. The primary antibodies and their dilutions were as follows: 1:1,000 DNA-PKcs (ab32566, Abcam), 1:1,000 NGAL (Abcam), 1:1,000 α-SMA(Abcam),1:500 γH2AX (9718S, Cell Signaling Technology), 1:1,000 Vimentin (5741S, Cell Signaling Technology) and 1:1,000 β-actin (4970S, Cell Signaling Technology). After incubation with horseradish peroxidase–conjugated secondary antibodies, the membranes were developed by using an enhanced chemiluminescence detection system. Protein expression was quantified on the basis of grey values by using Image-J software.

### 2.4. Immunofluorescence

The culture medium was removed. NRK-52E cells were washed with pre-cooled 1× PBS, added with pre-cooled 4% paraformaldehyde and fixed at 4 °C for 30 min. After 30 min of fixation, the cells were washed three times with pre-cooled 1× PBS. The cells were blocked with 0.3% TritonX-100 and 3% BSA at room temperature for 1 h and washed three times with 1× PBS in cold storage. Next, the cells were incubated with the primary antibody overnight at 4 °C. After incubation with the primary antibody, the cells were washed with 1× PBS three times. The secondary antibody was prepared. Alexa Fluor647–coupled secondary antibodies must be incubated under dark conditions. Therefore, the cells were incubated at room temperature in a wet box for 1 h. They were then washed three times with 1× PBS. Cell nuclei were stained with 4′,6-diamidino-2-phenylindole and washed three times with 1× PBS. Images were obtained by using laser scanning confocal microscopy.

### 2.5. Transmission electron microscopy

NRK-52E cells were fixed with 2.5% glutaraldehyde pre-cooled to 4 °C for 15 min. The cells were gently scraped, transferred to an EP tube, and allowed to stand at 4 °C overnight to settle to the bottom of the EP tube. Subsequently, the cells were fixed in 1% osmic acid for 2 h and dehydrated and embedded in a standard manner. Appropriate areas were selected. Ultrathin sections with a thickness of 50 nm were stained with lead citrate and uranyl acetate and observed by using a transmission electron microscope (Hitachi).

### 2.6. Cell proliferation analysis

Cell Counting Kit-8 (CCK-8, Absin, Shanghai, China) was used to detect cell proliferation. In accordance with the manufacturer’s instructions, cells were seeded into 96 well plates at appropriate densities. The 96-well plates were placed in an incubator at 37 °C and 5% CO_2_, and the cells were cultured for the appropriate duration. A total of 10 μL of CCK-8 solution was added to each well, and the cell culture plates were incubated for 2 h. Absorbance was detected at 450 nm by using a plate reader. Blank wells (containing culture media and CCK-8 reagent) and control wells (containing untreated cells, culture media and CCK-8 reagent) were also detected.

### 2.7. Analysis of cell cycle distribution

Propidium iodide (PI, 550825, BD Biosciences) staining was used for cell cycle analysis. After treatment, cells were washed twice with cold PBS, harvested and fixed with 70% ethanol at 4 °C overnight. Next, the cells were washed with cold PBS twice and stained with PI/RNaseA staining buffer. The cells were incubated in the dark for 15 min at room temperature and subjected to flow cytometry (CytoFLEX LX, Beckman).

### 2.8. Renal I/R model

To avoid the interference of estrogen in female rats on kidney function parameters (such as inflammatory factors and oxidative stress levels), this study used 6–10-week-old male Sprague-Dawley rats and induced bilateral ischemia/reperfusion (I/R) injury in the male rats, based on previously reported literature [18]. Briefly, the bilateral renal pedicles of the rats were exposed and clamped for 45 min to induce ischemia. The clamps were then released, and the animals were sacrificed 6 h, 24 h, 48 h, 72 h, 1 week, 2 weeks and 4 weeks later as indicated. Each group consisted of six rats. Rats that were not subjected to renal pedicle clamping were used as no-I/R rats. The animal study was ethically approved by the Ethics Committee of of Shanghai Children’s Hospital(SHCH-IACUC-2021-XMSB-36).

### 2.9. Immunohistochemistry

Renal tissues were prepared for tissue immunohistochemistry by following a routine procedure in the laboratory [19]. The primary antibody was anti-DNA-PKcs antibody (1:100, ab32566, Abcam). Immunohistochemical staining was observed in 10 areas (200×) randomly by using a microscopy imaging system (Leica Microsystems, Wetzlar, Germany).

### 2.10. Enzyme-linked immunosorbent assay (ELISA)

The amount of creatinine in the blood was measured according to the manufacturer’s protocol. Malondialdehyde (MDA) levels and superoxide dismutase activity in kidney tissue and in the homogenate of NRK-52E cells were evaluated using ELISA kits.The malondialdehyde (MDA) content detection kit and superoxide dismutase (SOD) activity detection kit was purchased from Servicebio (China), the Creatinine Assay Kit was purchased from Beckman coulter (USA).

### 2.11. Statistical analysis

All data were expressed as mean ± standard deviation. The data of different groups were compared by one-way analysis of variance, followed by Bonferroni’s correction for multiple comparisons. *p* < 0.05 was considered statistically significant. Statistical analysis was conducted by using GraphPad Prism software.

## 3. Results

### 3.1. Expression of DNA-PKcs in the renal ischemia/reperfusion (I/R) model

**Figure 1.**
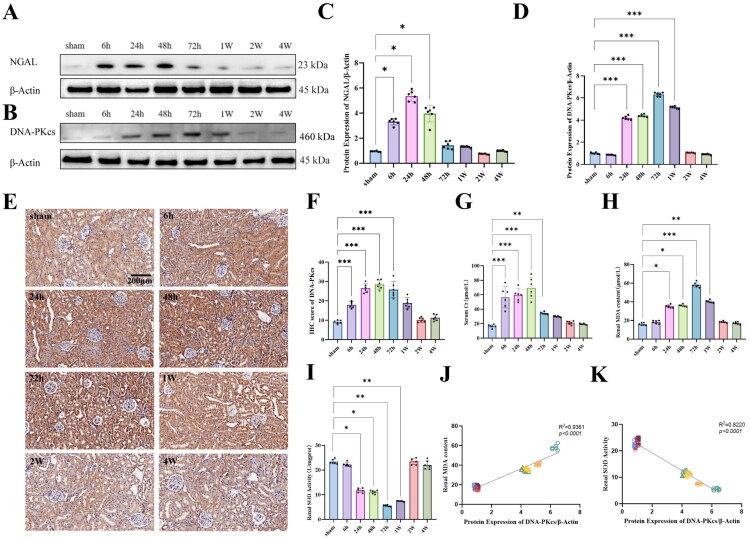
Expression of DNA-PKcs in the renal ischemia/reperfusion (I/R) model. (A) Representative pictures of the expression of NGAL detected by Western blot, β- actin is an internal parameter. (B) Representative pictures of the expression of DNA-PKcs detected by Western blot, β- actin is an internal parameter. (C) The expression of NGAL in rat I/R model at different times. (D) The expression of DNA-PKcs in rat I/R model at different times, n = 6. (E, F) Immunohistochemical representative pictures and statistical data of DNA-PKcs protein in rat I/R model at different times. Bar = 200μm. (G) Serum creatinine content at different times in rat I/R model, n = 6. (H) MDA content in kidney of rat I/R model at different times, n = 6. (I) SOD activity in kidney of rat I/R model at different times, n = 6. (J) Correlation analysis of protein expression level of DNA-PKcs and renal MDA content, n = 6. (K) Correlation analysis of protein expression level of DNA-PKcs and renal SOD activity, n = 6. Data are presented as means ± SD. *P < 0.05, **P < 0.01, ***P < 0.001.

To investigate the relationship between DNA-PKcs and renal injury, we established a rat ischemia/reperfusion (I/R) injury model. Animals were sacrificed at different time points following reperfusion, including 6 h, 24 h, 48 h, 72 h, 1 week, 2 weeks, and 4 weeks, and tissue samples were collected for analysis. Among the commonly used biomarkers of acute kidney injury (AKI), NGAL (neutrophil gelatinase-associated lipocalin) is one of the most frequently employed. Our results showed that NGAL expression was significantly elevated at 6 h post-reperfusion, peaked at 24 h, and gradually returned to baseline levels thereafter (Figure 1A and C). Additionally, serum creatinine levels were measured as an indicator of renal ischemia/reperfusion injury severity. Our results demonstrated a significant increase in serum creatinine at 6 h post-reperfusion, reaching its peak at 48 h, with a gradual normalization over the subsequent 4 weeks (Figure 1G). These findings indicate the successful establishment of a renal injury and repair model.

Since oxidative stress play an important role in the pathogenesis of I/R injury, we further assessed malondialdehyde (MDA) levels and superoxide dismutase (SOD) activity using ELISA. The results revealed that MDA levels significantly increased at 24 h post-reperfusion, peaked at 72 h, and gradually returned to normal within 2 weeks (Figure 1H). In contrast, SOD1 activity began to decline after 24 h, reaching a minimum at 72 h, and gradually recovered to baseline levels over the next 2 weeks (Figure 1I). These results further support the dynamic role of oxidative stress in both renal injury and repair.

Next, we assessed the expression of DNA-PKcs in the renal I/R model. Western blot analysis revealed a gradual increase in DNA-PKcs expression starting at 24 h post-reperfusion, peaking at 72 h, and returning to baseline levels over the subsequent 2 weeks (Figure 1B and D). Immunohistochemical staining indicated that DNA-PKcs was predominantly localized in renal tubular epithelial cells and its expression significantly upregulated during reperfusion (Figure 1E). Notably, between 6 h and 1 week post-reperfusion, DNA-PKcs was significantly elevated in both the nucleus. By 2 to 4 weeks post-reperfusion, DNA-PKcs levels in both compartments returned to baseline levels (Figure 1E and F).

It is noteworthy that the expression levels of DNA-PKcs were closely correlated with markers of oxidative stress. Specifically, protein expression of DNA-PKcs was significantly positively correlated with MDA levels and negatively correlated with SOD1 activity (Figure 1J and K). These findings suggest that DNA-PKcs play a role in the progression of kidney ischemia-reperfusion injury and its subsequent repair.

### 3.2. Expression of DNA-PKcs in the H/R NRK-52E cell model

**Figure 2.**
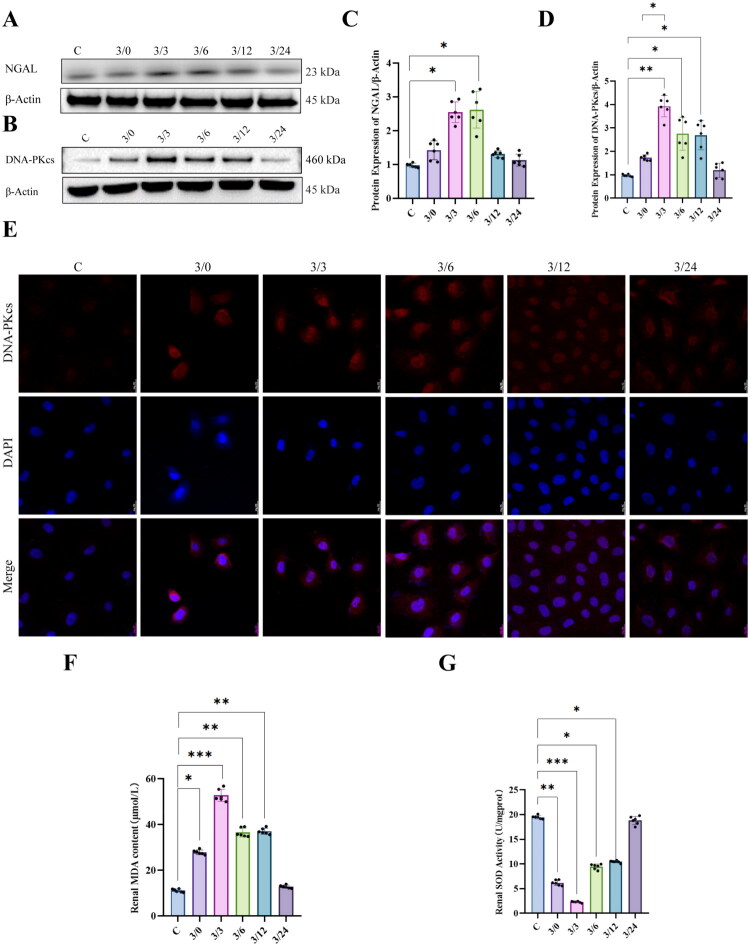
Expression of DNA-PKcs in the H/R NRK-52E cell model. (A) Representative pictures of the expression of NGAL detected by Western blot, β- actin is an internal parameter. (B) Representative pictures of the expression of DNA-PKcs detected by Western blot, β-actin is an internal parameter. (C) The expression of NGAL in H/R Model at different times. (D) The expression of DNA-PKcs in H/R Model at different times, n = 6. (E) The localization of DNA-PKcs in H/R model. DNA-PKcs: red fluorescent, DAPI: nucleus, Bar = 20μm. (F) MDA content in H/R model at different times, n = 6. (G) SOD activity in H/R model at different times, n = 6. Data are presented as means ± SD. *P < 0.05, **P < 0.01, ***P < 0.001.

To better investigate the mechanisms of I/R injury and its repair, we established a hypoxia/reoxygenation (H/R) model using NRK-52E cells. This model allows us to simulate oxygen deprivation during ischemic conditions at the cellular level, as well as the restoration of oxygen following reperfusion. In our previous studies, this model has been successfully used to replicate renal cell injury and the subsequent repair processes associated with ischemia/reperfusion [18]. Specifically, hypoxic conditions were induced by the application of mineral oil, followed by reoxygenation to simulate the repair process. As expected, we observed a sustained and significant increase in the expression of neutrophil gelatinase-associated lipocalin (NGAL) after 3 h of hypoxia, reaching a peak at 3 h post-reoxygenation, and subsequently returning to baseline levels within 24 h (Figure 2A and C). We further measured cellular malondialdehyde (MDA) levels *via* ELISA and found a sustained and significant increase in MDA content beginning at 3 h of hypoxia, peaking at 3 h of reoxygenation, and gradually declining back to baseline within 24 h (Figure 2F). Superoxide dismutase 1 (SOD1) activity, on the other hand, showed a sustained and significant reduction at 3 h of hypoxia, reached its nadir at 3 h of reoxygenation, and progressively recovered to baseline levels within 24 h (Figure 2G). These findings indicate that our model effectively mimics the damage and repair process of renal tubular epithelial cells driven by oxidative stress, with hypoxia causing significant injury and reoxygenation facilitating repair.

We next investigated the expression of DNA-PKcs using Western blot analysis (Figure 2B). The results showed a sustained and significant increase in DNA-PKcs expression following hypoxia, peaking at 3 h of reoxygenation, and subsequently decreasing, gradually returning to baseline levels within 24 h (Figure 2D). Immunofluorescence imaging revealed that DNA-PKcs exhibits a significant increase in cytoplasmic expression at 3 h of hypoxia. It subsequently relocates in large amounts to the nucleus at 3 and 6 h post-reoxygenation, and its levels gradually decrease at 12 and 24 h post-reoxygenation (Figure 2E). These results suggest that DNA-PKcs plays a crucial role in the repair of renal tubular epithelial cells following ischemia/reperfusion injury.

### 3.3. Effects of DNA-PKcs inhibition on H/R injury and repair in NRK-52E cells

**Figure 3.**
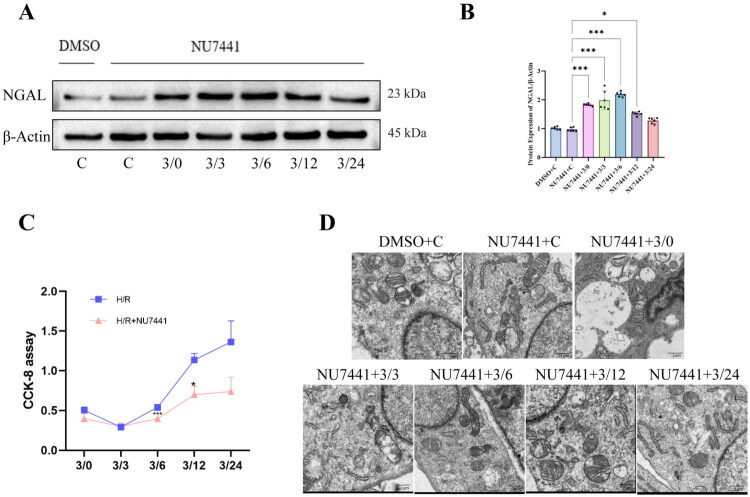
Effects of DNA-PKcs inhibition on H/R injury and repair in NRK-52E cells. (A) Western blot representative pictures of NGAL proteins, β- actin is an internal parameter. (B) The expression of NGAL in NRK-52E cells during H/R after inhibition of DNA-PKcs function. (C) Growth curve of NRK-52E cells with H/R and DNA-PKcs inhibition, n = 6. (D) Transmission electron microscopy results of NRK-52E cells inhibited by H/R and DNA-PKcs, n = 6. Yellow arrows point to damaged organelles, Bar = 1μm. Data are presented as means ± SD. *P < 0.05, **P < 0.01, ***P < 0.001.

NU7441 is a specific inhibitor of DNA-PKcs, and we employed the previously established hypoxia/reoxygenation (H/R) model to further investigate the role of DNA-PKcs in H/R-induced injury and subsequent repair in NRK-52E cells. Western blot analysis revealed that NGAL expression began to increase 3 h after hypoxia in combination with NU7441 treatment, reaching a peak at 6 h of reoxygenation, and gradually decreased thereafter until returning to baseline levels at 24 h of reoxygenation (Figure 3A and B). Cell viability, as assessed by CCK8 assay, demonstrated that the addition of NU7441 significantly impaired the recovery of cell viability at 6, 12, and 24 h of reoxygenation (Figure 3C).

To further examine the impact of DNA-PKcs inhibition on cellular morphology and structure, we performed transmission electron microscopy (TEM) analysis (Figure 3D). The control group treated with DMSO exhibited normal organelle morphology, with mitochondria showing distinct double membranes, smooth membrane surfaces, and no apparent swelling or degeneration in the inner matrix. The rough endoplasmic reticulum (RER) displayed a uniform distribution of ribosomes without noticeable swelling or degeneration, and the cell nucleus retained a normal shape with an intact nuclear membrane. The smooth endoplasmic reticulum (SER) and Golgi apparatus maintained well-preserved structures, and the plasma membrane was intact without evident protrusions or deformation. In contrast, the control group treated with the DNA-PKcs inhibitor (1 μM NU7441) showed mild mitochondrial swelling, with some mitochondria exhibiting irregular membrane deformation. The RER displayed significant swelling, and ribosome distribution was disrupted or reduced. The Golgi apparatus showed a less defined structure, indicating slight cellular damage caused by the DNA-PKcs inhibitor. Following hypoxia, cells exhibited severe damage, including pronounced mitochondrial swelling, significant membrane rupture, and increased fluid accumulation in the matrix. The RER was severely swollen, with blurred membrane structures and considerable ribosome detachment. The cell nucleus showed chromatin dispersion, and the nuclear membrane exhibited irregular changes. The Golgi apparatus further deteriorated, with swelling or disintegration. These findings suggest that hypoxia combined with DNA-PKcs inhibition exacerbates cellular damage in NRK-52E cells. After reoxygenation, the organelles gradually recovered over time, with mitochondria being the first to show repair signs, followed by the RER and Golgi apparatus. However, even at 24 h post-reoxygenation, minor organelle damage persisted.

The above-mentioned suggests that 1 μM of NU7441 may have delayed the recovery of cellular function after reoxygenation (Figures 2A, C and 3).

### 3.4. DNA-PKcs inhibition delays DNA damage repair in H/R-treated NRK-52E cells

The evidence above provides preliminary support for the involvement of DNA-PKcs in the repair of renal ischemia-reperfusion (I/R) injury; however, the specific mechanisms underlying this process remain unclear. Previous studies have demonstrated that DNA-PKcs plays a pivotal role in DNA damage repair, maintaining genomic stability, regulating the cell cycle, and triggering mitochondrial dysfunction. Consequently, we first aimed to investigate whether the involvement of DNA-PKcs in renal I/R injury repair is associated with DNA damage repair.

**Figure 4.**
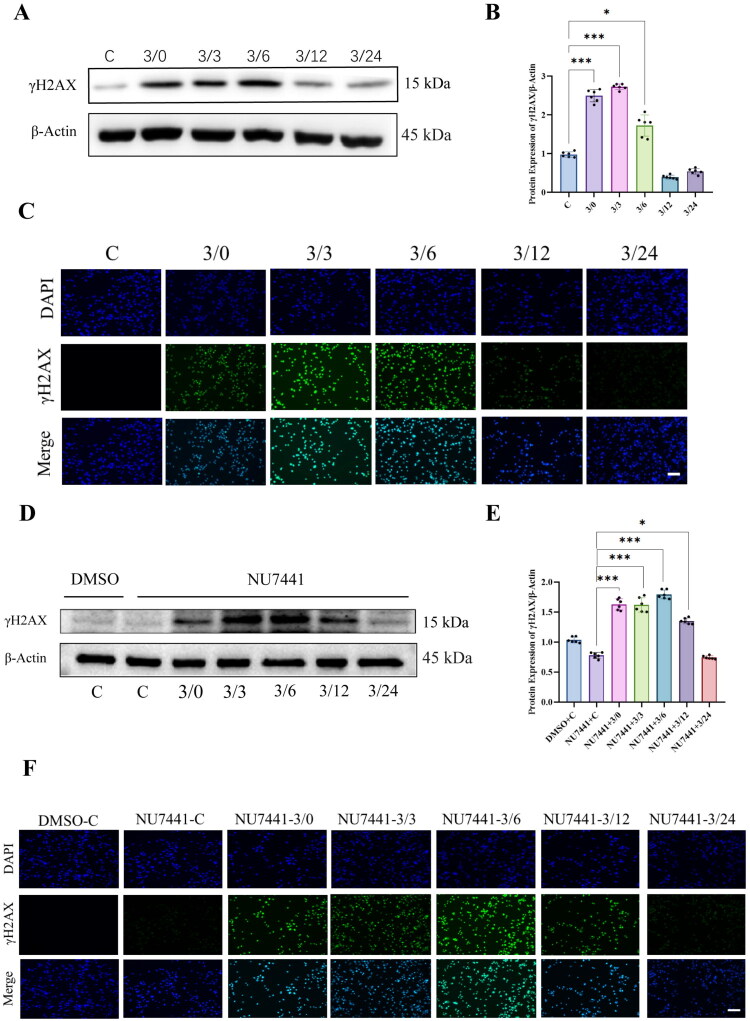
DNA-PKcs inhibition delays DNA damage repair in H/R-treated NRK-52E cells. (A) Representative Western blot images of γH2AX expression in NRK-52E cells during H/R, β-actin using as an internal parameter. (B) The expression of γH2AX in NRK-52E cells during H/R process, n = 6. (C) Representative immunofluorescence images of γ-H2AX expression in NRK-52E cells during H/R, scale bar = 50um. (D) Representative Western blot images of γH2AX expression after DNA-PKcs function was inhibited, β-actin using as an internal parameter. (E) The expression of γH2AX during NRK-52E cells hypoxia/reoxygenation after DNA-PKcs function was inhibited, n = 6. (F) Representative immunofluorescence images of γ-H2AX expression in NRK-52E cells during H/R after DNA-PKcs function was inhibited, scale bar = 50um.Data are presented as means ± SD. *P < 0.05, **P < 0.01, ***P < 0.001.

γ-H2AX is a recognized DNA damage marker. Our WB results show that γ-H2AX significantly increases after hypoxia, peaks 3 h after reoxygenation, and then returns to baseline levels 12 h after reoxygenation (Figure 4A and B). Immunofluorescence results show that the expression of γ-H2AX significantly increases after hypoxia and is localized in the nucleus. The level of γ-H2AX peaks at 3 h of reoxygenation and returns to baseline levels at 12 h of reoxygenation (Figure 4C). It should be emphasized that both WB and immunofluorescence experiments revealed that, following the inhibition of DNA-PKcs activity with NU7441, γ-H2AX levels significantly increased after 3 h of hypoxia, peaked at 6 h of reoxygenation, and returned to normal levels until 24 h. (Figure 4D–F). These findings indicate that inhibition of DNA-PKcs function prolongs the elevated expression of γ-H2AX after reoxygenation, suggesting that DNA-PKcs inhibition may lead to a sustained damage response in cells, thereby supporting the involvement of DNA-PKcs in DNA damage repair.

### 3.5. Inhibition of DNA-PKcs function affects the cell cycle in H/R-treated NRK-52E cells

**Figure 5.**
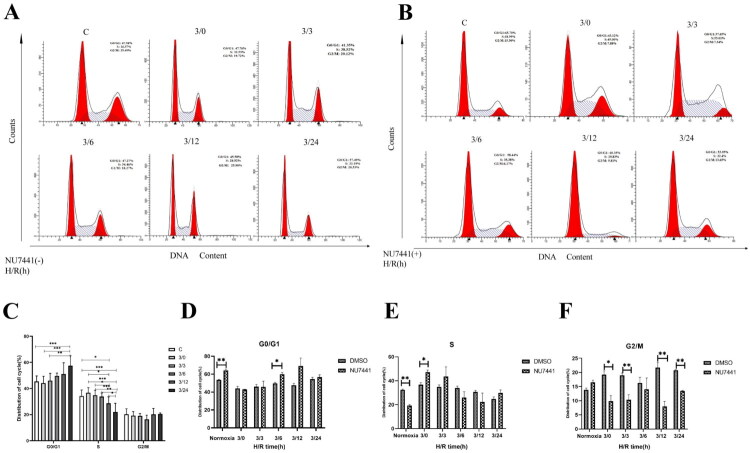
Inhibition of DNA-PKcs function affects the cell cycle in H/R-treated NRK-52E cells. (A) Representative diagram of cell cycle distribution in the H/R model of NRK-52E cells. (B) Representative diagram of cell cycle distribution in the H/R model after DNA-PKcs function inhibition. (C) Statistics of cell cycle distribution in the H/R model of NRK-52E cells. (D) G0/G1 phase change in the H/R model after DNA-PKcs function inhibition. (E) S phase change in the H/R model after DNA-PKcs function inhibition. (F) Change of G2/M phase in H/R model after DNA-PKcs function inhibition, n = 3. Data are presented as means ± SD. *P < 0.05, **P < 0.01, ***P < 0.001.

Subsequently, we utilized flow cytometry to analyze the impact of DNA-PKcs inhibition on the cell cycle progression (Figure 5A and B). Flow cytometric analysis revealed that following hypoxia/reoxygenation (H/R), the proportion of cells in the G0/G1 phase gradually increased, while the proportion of cells in the S phase decreased, with no significant changes observed in the G2/M phase (Figure 5C and D). This suggests that H/R inhibits the normal progression of the cell cycle. Upon inhibition of DNA-PKcs leads to a further increase in the proportion of G0/G1 phase cells and a decrease in the number of S phase cells 6 h after hypoxia-reoxygenation, resulting in a G1/S phase arrest. (Figure 5D and E). In addition, we also found that inhibition of DNA-PKcs leads to a decrease in the proportion of G2/M phase cells during both hypoxia and reoxygenation stages (Figure 5F). These findings suggest that the inhibition of DNA-PKcs disrupts the cell cycle during the repair process of renal tubular epithelial cells, impairing cell division and proliferation, thereby delaying the repair process following hypoxia/reoxygenation injury.

### 3.6. Inhibition of DNA-PKcs function affects epithelial-mesenchymal transition in H/R-treated NRK-52E cells

Renal ischemia-reperfusion (I/R) injury leads to structural damage in kidney tissue, tubular dilation, cellular detachment, and necrosis, gradually progressing to fibrosis [20]. Epithelial-mesenchymal transition (EMT) plays a pivotal role in the pathogenesis of renal fibrosis[21]. In rat models of renal ischemia-reperfusion injury, mitochondrial DNA damage induced by oxidative stress is associated with the occurrence of EMT[21]. To explore whether DNA-PKcs participates in the repair of renal ischemia-reperfusion injury and whether this process is linked to EMT in renal tubular epithelial cells, we employed the DNA-PKcs inhibitor NU7441 and subjected NRK-52E cells to a 24-h hypoxia/reoxygenation (H/R) treatment. Western blot analysis was performed to assess the expression levels of EMT markers, including α-SMA and Vimentin, during H/R.

**Figure 6.**
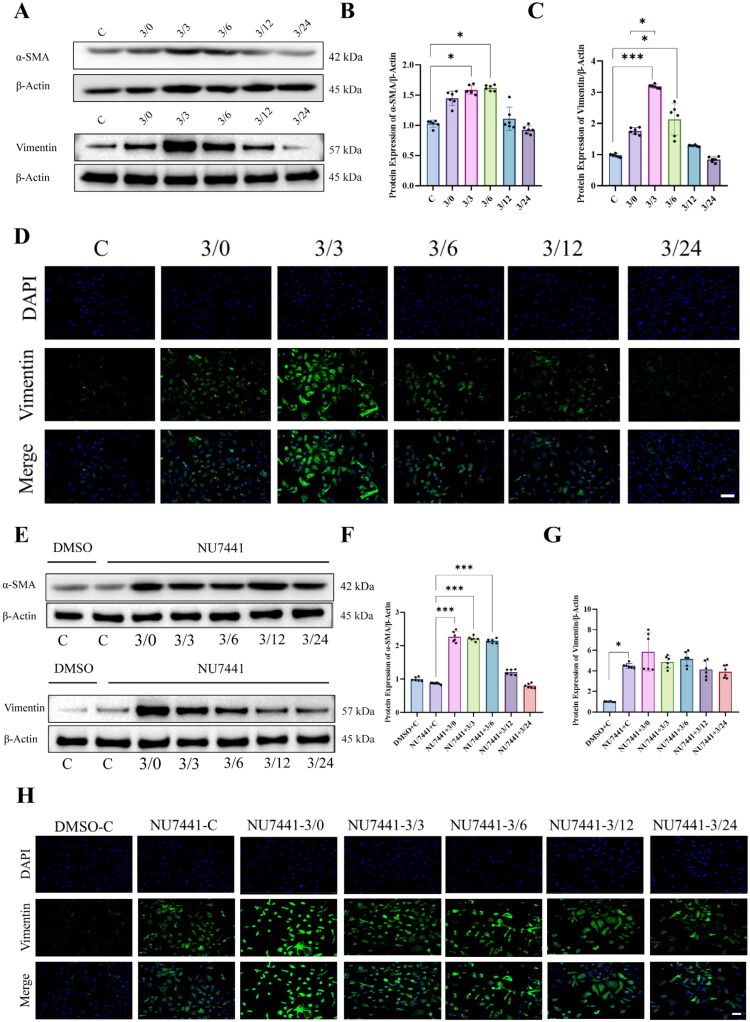
Inhibition of DNA-PKcs function affects epithelial-mesenchymal transition in H/R-treated NRK-52E cells. (A) Representative Western blot images of a-SMA and Vimentin expression in NRK-52E cells during H/R process, β-actin using as an internal parameter. (B) The expression of a-SMA in NRK-52E cells during H/R process, n = 6. (C) The expression of Vimentin in NRK-52E cells during H/R process, n = 6. (D) Representative IF images of the expression of Vimentin after DNA-PKcs function inhibition, β- actin is an internal parameter. (E) representative Western blot images of the expression of α-SMA and Vimentin after DNA-PKcs function inhibition, β-actin is an internal parameter. (F) After DNA-PKcs function was inhibited, the expression of α-SMA changes during cell H/R, n = 6. (G) After DNA-PKcs function was inhibited, the expression of Vimentin changes during cell H/R, n = 6. (H) Representative IF images of the expression of Vimentin after DNA-PKcs function inhibition. Data are presented as means ± SD. *P < 0.05, **P < 0.01, ***P < 0.001.

The Western blot results showed that the expression of α-SMA did not significantly increase during the hypoxic phase, but its level significantly increased after 3 h of reoxygenation, peaking at 6 h of reoxygenation, then gradually decreasing and returning to baseline levels within 24 h (Figure 6A and B). In addition, the expression of Vimentin peaked at 3 h of reoxygenation, then gradually decreased and returned to baseline levels within 24 h (Figure 6A and C), with similar results observed in immunofluorescence (IF) results (Figure 6D). After inhibiting DNA-PKcs function with NU7441, α-SMA expression significantly increased at 3 h of hypoxia and remained at a high level until 6 h of reoxygenation, then gradually returned to baseline levels within 24 h (Figure 6E and F). Both WB and IF revealed that inhibition of DNA-PKcs function leads to an increase in Vimentin expression under normoxic conditions, and Vimentin expression remains at a higher level during both hypoxia and reoxygenation, with no significant decrease observed (Figure 6E, G and H). These findings suggest that EMT occurs during the repair process of renal tubular epithelial cells following H/R injury, and that DNA-PKcs plays a crucial role in regulating this transition. The effects of NU7441 on Vimentin expression were more pronounced than those on α-SMA, indicating that DNA-PKcs is vital in modulating the dynamic regulation of EMT to support tubular repair.

## 4. Discussion

Renal ischemia/reperfusion (I/R) injury is a common cause of acute kidney injury (AKI) [22]. Ischemia/reperfusion (I/R) injury can lead to the production of large amounts of reactive oxygen species (ROS) by renal tubular epithelial cells (TECs), which in turn induces mitochondrial damage, lipid peroxidation, and DNA fragmentation, resulting in catastrophic cellular damage [23,24]. Given the critical role of DNA-dependent protein kinase catalytic subunit (DNA-PKcs) in DNA damage repair [25], we hypothesized that DNA-PKcs is essential for repairing AKI-induced damage.

To investigate this, we first established an *in vivo* renal I/R model in rats. Analysis of hypoxic injury markers, I/R injury severity, and DNA-PKcs expression across multiple reperfusion time points revealed a close association between DNA-PKcs dynamics and the I/R injury/repair process. Subsequently, we utilized an *in vitro* hypoxia/reoxygenation (H/R) model, widely employed in I/R research, to further explore the role of DNA-PKcs in the repair of renal tubular cell damage induced by hypoxia and reoxygenation [26]. While H/R-induced damage is well-studied, the subsequent repair phase remains less explored. Therefore, we extended the observation period following reoxygenation to better assess the changes in cellular functions during the repair phase. In our model, we observed that DNA-PKcs accumulates following H/R-induced damage and gradually returns to baseline levels during the repair phase, which is consistent with our findings in animal experiments. Therefore, *in vivo* and *in vitro* experiments suggest that DNA-PKcs may be involved in the repair process of I/R.

To further investigate the role of DNA-PKcs in the repair process, we used the highly efficient and selective DNA-PK inhibitor NU7441, which effectively inhibits DNA-PKcs function at relatively low doses. Inhibition of DNA-PKcs significantly delayed the repair of renal tubular epithelial cells and reduced cell viability during the repair phase after injury. Ultrastructural analysis revealed that, although there was substantial recovery of organelles such as the endoplasmic reticulum and mitochondria after 24 h of repair compared to the early stages of hypoxic injury repair (3h and 6h of reoxygenation), slight damage still remained. Inhibition of DNA-PKcs leading to sustained DNA damage, interruption of cell cycle progression, reduced cell proliferation, and enhanced EMT. Specifically, DNA-PKcs has been shown to play a role in DNA damage repair, as evidenced by the transient upregulation of γH2AX expression in the nucleus and its subsequent resolution. Inhibition of DNA-PKcs prolonged the persistence of DNA damage, highlighting its critical role in timely repair. This finding is consistent with previous studies demonstrating the role of DNA-PKcs in promoting non-homologous end joining (NHEJ) repair [7–9]. Additionally, DNA-PKcs regulates cell cycle dynamics by promoting progression from the G1 phase to the S phase and supporting mitotic activity. Functional deficiency of DNA-PKcs leads to cell cycle arrest and decreased cell proliferation, further impairing the repair process. These results align with studies emphasizing the role of DNA-PKcs in regulating cell cycle checkpoints and facilitating mitotic progression [13,27]. EMT, a key adaptive response to tubular injury, is also influenced by DNA-PKcs, with its inhibition leading to sustained and aggravated expression of EMT markers such as α-SMA and vimentin. This suggests that DNA-PKcs regulates partial EMT (pEMT), which serves as a critical determinant in the transition from adaptive to maladaptive repair [28,29].

In conclusion, our findings establish DNA-PKcs as a pivotal regulator of AKI repair. It facilitates the recovery of I/R-injured TECs by orchestrating DNA damage repair, maintaining cell cycle progression, supporting proliferation, and preventing aberrant EMT. Inhibition of DNA-PKcs significantly impairs these repair mechanisms; therefore, DNA-PKcs represents a potential therapeutic target for this disease. However, a primary limitation of this study is the absence of direct interventions (e.g. gene knockdown or pharmacological inhibition) in the *in vivo* ischemia-reperfusion (I/R) disease model. This deficiency significantly limits our ability to definitively establish DNA-PKcs’ causal role within complex physiological environments and assess its therapeutic potential. Future investigations should validate these findings through two approaches: employing conditional knockout models during I/R injury repair phases, or administering DNA-PKcs inhibitors (such as NU7441) *in vivo*. Another important limitation of the present study is that only male animal models were used; female animals were not included. Given that this disease affects both sexes, future studies should incorporate female animals to enhance the generalizability of the conclusions. In summary, follow-up work will be dedicated to further elucidating the therapeutic potential of DNA-PKcs in AKI.

## Data Availability

The data that support the findings of this study are available from the corresponding author on reasonable request.
